# Gender-Specific Body Composition Relationships between Adipose Tissue Distribution and Peak Bone Mineral Density in Young Chinese Adults

**DOI:** 10.1155/2020/6724749

**Published:** 2020-04-01

**Authors:** Zeyu Xiao, Hao Xu

**Affiliations:** ^1^Molecular Imaging Institute, The First Affiliated Hospital, Jinan University, Guangzhou, Guangdong, China; ^2^Department of Radiology, The First Affiliated Hospital of Jinan University, Guangzhou, Guangdong, China; ^3^Department of Nuclear Medicine, The First Affiliated Hospital of Jinan University, Guangzhou, Guangdong, China

## Abstract

**Background:**

The relationships between adipose tissue distribution and peak bone mineral density (BMD) in young adults are still unclear. The aim of this study was to investigate the body composition associations between fat mass (FM), lean mass (LM), regional adipose tissue distribution, and peak BMD across a cohort of young Chinese adults.

**Methods:**

Dual-energy X-ray absorptiometry (DXA) scans were performed on 786 men and 825 women aged from 20 to 40 years old to measure the markers for whole-body LM, FM, and BMD in the lumbar spine (LS), femoral neck (FN), and total hip (TH) areas. The android/gynoid FM ratio (A/G FMR) based on the DXA scans was calculated as an indicator of adipose tissue distribution. Pearson's correlation and multivariable linear regression analyses were conducted to determine the body composition relationships between adipose tissue distribution and BMD of each skeletal site.

**Results:**

The body composition characteristics were different in young males and females: a higher body FM percentage was found in females, while males had higher LM and A/G FMR. The markers for WBLM and WBFM had significant positive correlations with BMD among the linear regression analyses in both genders, while the relationships between A/G FMR and BMD were different in males and females; significant inverse associations were showed in all skeletal sites for women (standard *β* ranged from -0.266 to -0.170, *P* < 0.001 for all), but no significant relationships were found in men except for an inverse association in the LS skeletal site (standard *β* with -0.115, *P* = 0.016).

**Conclusions:**

In this sample of young Chinese adults, both whole-body lean mass and fat mass had significant positive relationships with BMD in both genders. The A/G FMR, as an indicator of central adipose tissue distribution, was inversely associated with BMD, especially in females.

## 1. Introduction

Body composition is an important determinant of bone mineral density (BMD) [1, 2]. Body composition consists primarily of two components: lean mass (LM) and fat mass (FM), which may directly affect bone or influence it indirectly as a mediator [3]. Numerous studies have explored the relationship between whole-body composition and BMD, in which total LM has consistently been shown to be positively correlated with BMD [4]. In addition, muscle and bone have common genetic, lifestyle, and hormonal determinants [5].

The underlying heterogeneous relationship between fat and bone may be attributed to different regional fat depots, such as subcutaneous and visceral fat, which have reported differences in both histology and metabolic activity. Visceral fat, regarded as an endocrine organ, is considered a detrimental factor in cardiovascular and cerebrovascular diseases [6]. Previously, several studies have indicated that different fat depots may have distinct relationships with bone [7]. Wang et al. reported that subcutaneous fat area is negatively associated with BMD in postmenopausal females [8]. A positive association between subcutaneous adipose tissue and bone density has been reported [9]. There are also conflicting data regarding the association between visceral AT and bone [10–12]. These controversial findings may result from a small sample size or different ethnicities in their studies. In light of this, we restudied the relationship of body composition, adipose tissue distribution, and BMD with a large sample size.

In a previous study, it was reported that different body composition was found in men and women, with higher lean mass in males and higher fat mass in females in a certain body mass index (BMI). Furthermore, the fat was mainly distributed in the trunk in males, while the fat tended to be deposited in the limbs and hips in females, particularly in the lower body [7]. Android versus gynoid and masculine versus feminine are always used to define the central versus peripheral fat distribution [13]. Zhang et al. [14] also reported that more visceral adipose tissue and less subcutaneous adipose tissue measured with computerized tomography scans were observed in men than women. However, most studies have mainly concentrated on a large population of healthy people without consideration of the influence of whole-body LM and FM on bone mass, for which the relationship with BMD may be different, especially in obese people with a higher body weight.

Our previous study demonstrated that the changes of body composition including LM and FM were age- and sex-related [15]. Regarding young adults aged from 20 to 40 years old, studies have indicated that their skeletal system will reach the peak bone mass in this range. Peak BMDs are important predictors to evaluate skeletal maturity and avoid bone loss and subsequent bone fractures. However, there have been few studies reporting the relationships of fat distribution with peak BMD in young adults, while most previous studies have mainly concentrated on adolescents or middle-aged and elderly people, especially postmenopausal women [4, 16].

Dual-energy X-ray absorptiometry (DXA), which can be used to measure both regional and whole-body mass, has been widely employed to evaluate body composition [17, 18]. In the current study, we aimed to investigate the body composition associations between fat mass (FM), lean mass (LM), regional adipose tissue distribution, and peak BMD, using DXA in a large population-based sample of Southern Chinese adults aged from 20 to 40 years old.

## 2. Materials and Methods

### 2.1. Participants

We recruited a cohort of young adults aged from 20 to 40 years old from the First Affiliated Hospital of Jinan University between January 2007 and December 2017. Our recruitment criteria were as follows: subjects are functionally independent Chinese individuals over 20-40 years of age, who were in apparent good health. Subjects were included if they did not meet any of the following exclusion criteria: (1) had a history of fractures in the lumbar spine (LS), femoral neck (FN), and/or hip bone; (2) took medication known to alter bone metabolism, body composition, and weight or fat distribution (such as estrogens, androgens, thyroid hormones, corticosteroids, or other related drugs); (3) suffered from chronic systemic diseases known to alter bone mass or body weight (such as hyperthyroidism, thyrotoxicosis, hyperparathyroidism, rheumatoid arthritis, chronic renal failure, or malignancy); and/or (4) were young women who experienced irregular menstruation or menopause due to pregnancy or polycystic ovarian syndrome. Medical histories of illness, medication use, smoking status, alcohol consumption, and female menstrual status were collected through standardized questionnaires. In this study, smokers were defined as those who smoked at the time of the study or who had a smoking history within 5 years. Those who had stopped smoking for 5 years or more were regarded as nonsmokers. Regular alcohol consumers were defined as those who consumed alcohol at least three times per week [9]. Eventually, 786 men and 825 women were included in our study. The study was approved by the Ethics Committee of the First Affiliated Hospital of Jinan University (Ethical Application Ref: 19017). All of the subjects provided informed written consent prior to participating in the study.

### 2.2. Basic Characteristics and Body Composition Measurements

A research physician obtained information on the medical history and medication use of each participant through personal interviews. Height and body weight were obtained based on standard methods: height was measured without shoes to the nearest 0.1 cm, and weight was measured while only wearing light clothing to the nearest 0.1 kg. Height was measured using a wall-mounted stadiometer (error range in 5 mm), and weight was measured using a digital scale (error range in 0.01 kg). Both parameters were measured twice, and the average of the two measurements was used. The mean variable coefficient (CV) of height was 0.63% ± 0.24% and weight was 0.78% ± 0.55%. Whole-body LM, FM, and BMD were obtained by whole-body scan, and BMD in the LS, FN, and total hip (TH) skeletal sites were measured by independent regional DXA scans (Lunar Prodigy, GE Healthcare, Madison, WI, USA), after which the data were analyzed using software version 10.0 provided by the manufacturer (the android and gynoid regions were automatically examined). From these measurements, BMI was calculated as weight/height^2^, FM index (FMI) as total FM/height^2^, LM index (LMI) as LM/height^2^, body FM% as total body FM/weight × 100%, and android/gynoid FM ratio (A/G FMR) as android FM/gynoid FM. Daily quality assurance scans were performed by scanning a spine phantom according to the manufacturer's instructions. All DXA measurements were conducted by the same trained technologist throughout the study. The CV was 1.3% ± 0.58% for whole-body LM, 1.74% ± 0.38% for FM, 1.8 ± 0.79% for regional LS BMD, 1.3 ± 0.75%for FN BMD, and 1.52% ± 0.46% for TH BMD, and the long-term reproducibility of the DXA varies over a small range.

### 2.3. Statistical Analysis

The normal distributions of the examined features were evaluated using the Kolmogorov–Smirnov test. *P* > 0.05 was considered to obey normal distribution. The continuous measures are expressed as mean ± standard deviation (SD). The mean differences of the baseline characteristics between males and females were compared using two independent-sample *t*-tests or chi-squared tests, and the Bonferroni corrections were also applied. Pearson's correlation analysis was conducted to calculate the correlation strength between independent variables and each regional BMD. A multivariable linear regression model was used to assess the relationships between A/G FMR and BMD using age, smoking status, and alcohol consumption as covariates and whole-body LM and FM as independent variables, and we chose the enter methods and expressed the results as standard *β* coefficients. All tests were two-tailed, and a *P* value of <0.05 was considered statistically significant. All statistical analyses were performed using the Statistical Package for the Social Sciences (Version 19.0) (SPSS Inc., Chicago, IL, USA); and GraphPad Prism 7.0 (GraphPad Software, California, USA) was used for graphic analysis.

## 3. Results

### 3.1. Basic Characteristics

Details of the basic characteristics are reported in Table 1. The mean age of the male subjects was 32.5 ± 5.5 years, and that of the female subjects was 30.7 ± 6.1 years. The males were significantly heavier and taller and had a higher BMI than the females. Regarding the body composition measurements, males had higher whole-body LM, LMI, A/G FMR, and trunk/leg FMR, while higher whole-body FM, FMI, and body FM% were found in females. In view of the BMD measurements, higher values were seen for the whole body and FN in young men, but not in the LS and TH. The epidemiological data revealed that more current smokers and alcohol users were male.

### 3.2. Correlation Analyses between Body Composition and BMD

The correlation coefficients between age, height, weight, BMI, body composition, and each BMD aspect are provided in Table 2. The correlation analyses suggest that weight, height, BMI, whole-body LM and FM, and body FM% had positive correlations with the whole-body, LS, FN, and TH BMDs in both males and females. The results also revealed that age was positively correlated with whole-body and each BMD area in females and whole-body BMD in males. However, a negative correlation was observed between age and FN BMD in males. A/G FMR showed positive correlations with whole-body, LS, FN, and TH BMD in males but not in females.

### 3.3. Multivariable Linear Regression Analyses for BMD

**Figure 1:**
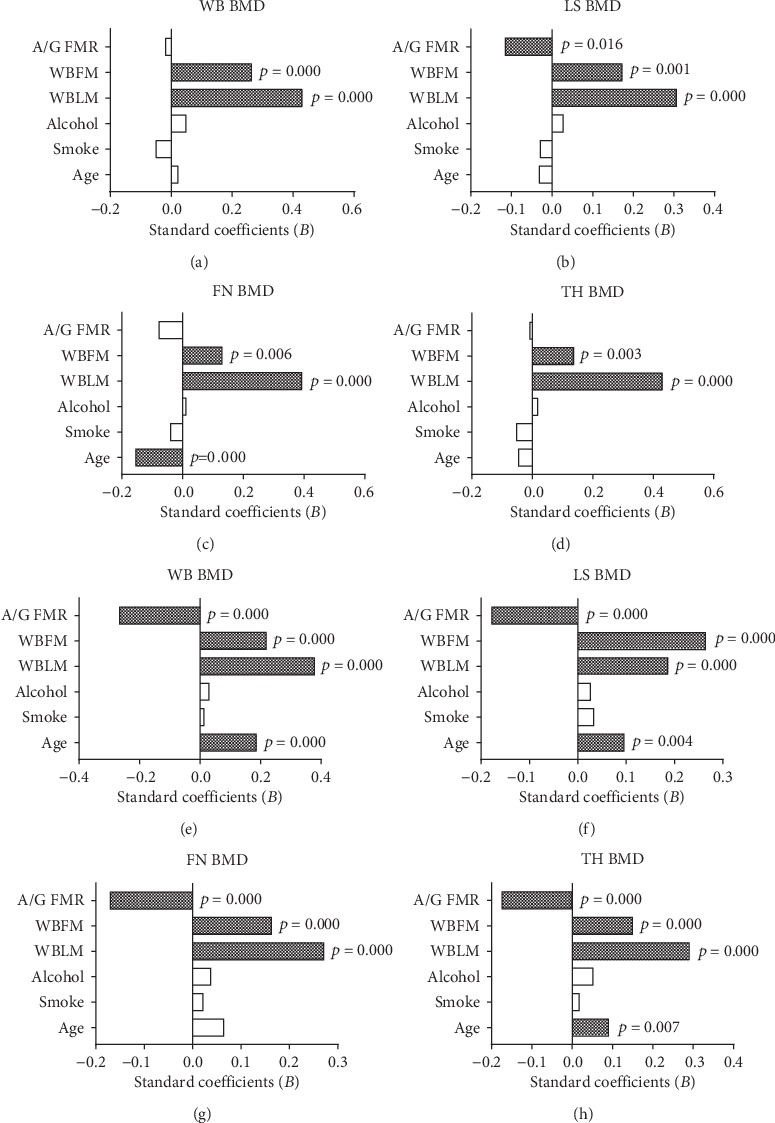
The standard *β* coefficients of bone mineral density at different skeletal sites with age, smoke, alcohol, WBLM, WBFM, and A/G FMR by the multiple regression analyses in males (a–d) and females (e–h). WBLM: whole-body lean mass; WBFM: whole-body fat mass; A/G FMR: android/gynoid fat mass ratio; WB BMD: whole-body bone mineral density; LS BMD: lumbar spine bone mineral density; FN BMD: femoral neck bone mineral density; TH BMD: total hip bone mineral density.

To further explore the independent predictive value of A/G FMR and whole-body composition for BMD at all skeletal sites in both genders, covariates such as age, smoking, and alcohol consumption were also included in the multiple linear regression analyses, and the standardized regression coefficients (*β*) and *t* and *P* values are listed in Table 3 and Figure 1. The effect sizes of A/G FMR and whole-body LM and FM on BMD were different according to the skeletal site and gender. Both whole-body LM and FM had significant and positive relationships with BMD in males and females, and whole-body LM was the strongest predictor in males (standard *β* ranged from 0.306 to 0.429, *P* < 0.001 for all), while whole-body FM seems more relatively associated with BMD in females (standard *β* ranged from 0.163 to 0.264, *P* < 0.001 for all). Interestingly, A/G FMR showed significant inverse associations that were found in all skeletal sites for women (standard *β* ranged from -0.266 to -0.170, *P* < 0.001 for all), but no significant relationships were found in males except for an inverse association in the LS skeletal site (standard  *β* with -0.115, *P* = 0.016). In the linear regression models, age was slightly positively related with BMD in females except for the FN skeletal site.

## 4. Discussion

The results of the present study provide an insight into the body composition relationships for different skeletal site BMDs in young adults aged from 20 to 40 years old. We have performed multivariable regression analyses to confirm whether A/G FMR was an independent predictor for BMD after adjusting for age, smoking status, alcohol consumption, and whole-body LM and FM. However, the results were not completely consistent with previous reports on adolescents [19, 20] and the elderly [10, 21].

In this study, males were heavier and had a higher BMI than females in this particular age group, the higher weight mainly arising from an increase in LM and decrease in FM, as revealed by the composite measures. Women had higher whole-body FM and body FM% and lower A/G FMR compared with men. To explore the influence of body composition on BMD, several bone regions were measured, and meaningful relationships were found between them. In this study, the body weight still had a closer relationship with BMD in both genders but also whole-body LM, FM, body FM%, and regional adipose tissue distribution. Based on this, we separated whole-body LM and FM from body weight and found that whole-body LM had a closer relationship with BMD than whole-body FM. A previous study indicated that BMI seems to be more closely associated with FM as each additional kilogram of FM is correlated with a two- to three-fold greater increase in BMI than each additional kilogram of LM, which indicates that LM may be a stronger predictor than FM for BMD measures [22]. The impact of mechanical loading (mainly arising from LM) on increasing bone density decreases as BMI increases in females, indicating that FM acting on bone mass may undergo a different mechanism [23].

Previous studies have suggested that in addition to the gravitational loading of FM on BMD, adipose tissue can also secrete multiple hormones such as leptin, insulin, adiponectin, and adipocytic estrogens and thus affect bone metabolism via endocrine mechanisms [24]. These hormones, which are identified as protective factors against the development of osteoporosis, help to promote the differentiation of osteoblasts and downregulate bone resorption [25]. These results are only partly consistent with our findings, which could have been due to the younger age of our participants, who would therefore have had high levels of hormone synthesis and secretion activity, as was especially seen in the males.

A previous study has suggested that visceral adipose tissue was inversely associated with BMD, and having more subcutaneous adiposity than visceral adipose tissue tends to be beneficial to skeletal health after the menopause [9], while both subcutaneous and visceral adipose tissue showed negative associations with BMD in the study of Katzmarzyk et al. [24]. These inconsistent findings stimulated our curiosity regarding whether there is different bone mass or bone metabolism due to a difference in fat distribution in the young adults in this study, in which we found that A/G FMR was an independent negative predictor of BMD after adjusting for body composition and BMI. However, the relationship between A/G FMR and BMD was not completely consistent for males and females. We divided the body adipose tissue into android fat representing the visceral (trunk) adipose tissue and gynoid fat reflecting the subcutaneous (leg) adipose tissue. Our results suggest that central adipose tissue accumulation could impair bone mineral maintenance and bone mass increase. After activating osteoclasts and inhibiting osteoblasts, bone resorption increases, which exerts a deleterious effect on skeletal stability and can precipitate osteopenia and osteoporosis [26]. Meanwhile, regional fat mass distribution had a more extensive effect on whole-body BMD in females rather than males. These results seem to imply that in young adults, hormone-related adipose tissue plays a leading role in general bone mass increase in females and mechanical loading-related lean muscle seems to be more important in regional bone formation in males, although differences in mechanical loading across different sites may alter the aforementioned relationships.

Several limitations should be mentioned regarding this study. First, no blood samples were obtained from the participants, and thus, the true hormone and cytokine levels were unknown. The potential mechanisms acting on bone mass for our data and the statistical results were mainly referenced from previous reports. Second, we only collected cross-sectional data and could not directly draw conclusions regarding the causality due to the limited design of our study. Third, although we evaluated the body composition relationships between regional adipose tissue distribution and BMD by adjusting for age, smoking status, and alcohol consumption, other confounders such as socioeconomic status, dietary intake, and physical activity, which may influence bone nutrition and metabolism, were not considered to be covariates in the multivariable regression analyses. Fourth, the measurement of the regional fat distribution was conducted by the DXA, while the accurate method is to obtain the volume of subcutaneous and visceral fat tissue by MRI or CT. Fifth, this was not a multicenter study as the participants mainly came from Southern China and thus cannot represent the general situation of young adults in China. Last, we had not stratified smoking status and alcohol consumption into more grades, which may have important effects on BMD and body composition in the ontogenesis range of 20-40 years.

## 5. Conclusions

In conclusion, females may have a higher body FM percentage and males have a higher LM and A/G FMR in the generation aged from 20 to 40 years old. We also found WBLM and WBFM had significant positive correlations with BMD in both genders. The A/G FMR, as an indicator of central adipose tissue distribution, was inversely associated with BMD in all skeletal sites in females in this sample of young Chinese adults, but A/G FMR only showed an inverse association with BMD in the LS skeletal site in males. Further research is required to explore the mechanisms underlying these findings, which may have public health implications for fracture prevention strategies in the context of the global obesity epidemic.

## Figures and Tables

**Table 1:** Baseline characteristics of subjects.

	Male	Female	*P* value
No. of subjects	786	825	
Age (years)	32.5 ± 5.5	30.7 ± 6.1	<0.001
Weight (kg)	63.9 ± 12.4	51.0 ± 9.0	<0.001
Height (cm)	168.8 ± 5.8	158.0 ± 5.0	<0.001
BMI (kg/m^2^)	22.4 ± 3.9	20.4 ± 3.2	<0.001
Body composition measures			
Whole-body LM (kg)	47.6 ± 6.0	33.0 ± 4.1	<0.001
LMI (kg/m^2^)	16.7 ± 1.7	13.2 ± 1.4	<0.001
Whole-body FM (kg)	13.7 ± 7.6	15.8 ± 6.1	<0.001
FMI (kg/m^2^)	4.8 ± 2.6	6.3 ± 2.3	<0.001
Fat%	20.2 ± 8.3	30.2 ± 6.8	<0.001
A/G FMR	0.57 ± 0.17	0.40 ± 0.10	<0.001
Trunk/legs FMR	1.99 ± 0.62	1.43 ± 0.33	<0.001
Bone mineral density (g/cm^2^)			
Whole body	1.105 ± 0.109	1.068 ± 0.918	<0.001
Lumbar spine	1.114 ± 0.162	1.118 ± 0.145	0.588
Femoral neck	0.921 ± 0.140	0.905 ± 0.123	0.019
Total hip	0.941 ± 0.150	0.928 ± 0.131	0.078
Current smoker (%)	18%	4%	<0.001
Current alcohol user (%)	15%	3%	<0.001

Note: values are presented as number, mean ± standard deviation, or percentage. LM: lean mass; LMI: lean mass index; FM: fat mass; FMI: fat mass index; Fat%: percentage of body fat mass; A/G FMR: android to gynoid fat mass ratio. *P* value was determined by the unpaired-sample *t*-tests or chi-squared test.

**Table 2:** Pearson's correlation between BMD at different skeletal sites with other parameters in the male and female groups.

	WB BMD	LS BMD	FN BMD	TH BMD
Males (*n* = 786)				
Weight	0.610^∗∗∗^	0.358^∗∗∗^	0.394^∗∗∗^	0.495^∗∗∗^
BMI	0.547^∗∗∗^	0.294^∗∗∗^	0.314^∗∗∗^	0.443^∗∗∗^
WBLM	0.567^∗∗∗^	0.355^∗∗∗^	0.423^∗∗∗^	0.500^∗∗∗^
WBFM	0.489^∗∗∗^	0.258^∗∗∗^	0.266^∗∗∗^	0.363^∗∗∗^
Fat%	0.407^∗∗∗^	0.203^∗∗∗^	0.182^∗∗∗^	0.280^∗∗∗^
A/G FMR	0.318^∗∗∗^	0.096^∗∗^	0.089^∗^	0.226^∗∗∗^
Trunk/legs FMR	0.401^∗∗∗^	0.216^∗∗∗^	0.164^∗∗∗^	0.321^∗∗∗^
Age	0.101^∗∗∗^	**-0.109**	-0.127^∗∗∗^	**0.017**
Height	0.306^∗∗∗^	0.245^∗∗∗^	0.274^∗∗∗^	0.232^∗∗∗^
Females (*n* = 825)				
Weight	0.424^∗∗∗^	0.339^∗∗∗^	0.315^∗∗∗^	0.319^∗∗∗^
BMI	0.369^∗∗∗^	0.305^∗∗∗^	0.264^∗∗∗^	0.295^∗∗∗^
WBLM	0.450^∗∗∗^	0.286^∗∗∗^	0.322^∗∗∗^	0.338^∗∗∗^
WBFM	0.276^∗∗∗^	0.269^∗∗∗^	**0.212**	0.206^∗∗∗^
Fat%	0.092^∗∗^	0.162^∗∗∗^	0.088^∗^	0.074^∗^
A/G FMR	**-0.049**	**0.004**	**-0.024**	**-0.028**
Trunk/legs FMR	0.088^∗^	0.152^∗∗∗^	**0.061**	0.102^∗∗^
Age	0.190^∗∗∗^	0.092^∗∗^	0.070^∗^	0.096^∗∗^
Height	0.271^∗∗∗^	0.198^∗∗∗^	0.237^∗∗∗^	0.166^∗∗∗^

Note: results expressed as *r* coefficients. WBLM: whole-body lean mass; WBFM: whole-body fat mass; Fat%: percentage of body fat mass; A/G FMR: android to gynoid fat mass ratio; WB BMD: whole-body bone mineral density; LS BMD: lumbar spine bone mineral density; FN BMD: femoral neck bone mineral density; TH BMD: total hip bone mineral density. The digits in bold denote a *P* value > 0.05. ^∗^*P* < 0.05, ^∗∗^*P* < 0.01, and ^∗∗∗^*P* < 0.001.

**Table 3:** Multiple regression analyses of bone mineral density at different skeletal sites with age, smoke, alcohol, WBLM, WBFM, and A/G FMR.

	WB BMD			LS BMD			FN BMD			TH BMD		
	Standard *β*	*t*	Sig.	Standard *β*	*t*	Sig.	Standard *β*	*t*	Sig.	Standard *β*	*t*	Sig.
Males												
Age	0.022	0.711	0.477	-0.031	-0.852	0.395	-0.154	-4.405	0.000	-0.045	-1.344	0.179
Smoke	-0.050	-1.726	0.085	-0.029	-0.860	0.390	-0.040	-1.243	0.214	-0.052	-1.677	0.094
Alcohol	0.048	1.675	0.094	0.027	0.793	0.428	0.011	0.332	0.740	0.017	0.545	0.586
WBLM	0.429	12.601	0.000	0.306	7.715	0.000	0.392	10.323	0.000	0.429	11.678	0.000
WBFM	0.263	6.229	0.000	0.172	3.488	0.001	0.129	2.740	0.006	0.136	2.978	0.003
A/G FMR	-0.019	-0.469	0.639	-0.115	-2.421	0.016	-0.077	-1.680	0.093	-0.001	-0.026	0.979
Females												
Age	0.185	6.057	0.000	0.095	2.849	0.004	0.064	1.921	0.055	0.089	2.681	0.007
Smoke	0.012	0.388	0.698	0.033	1.004	0.316	0.021	0.644	0.520	0.017	0.520	0.603
Alcohol	0.029	0.963	0.336	0.026	0.788	0.431	0.037	1.128	0.259	0.051	1.567	0.117
WBLM	0.378	11.132	0.000	0.186	4.993	0.000	0.271	7.266	0.000	0.290	7.846	0.000
WBFM	0.219	5.788	0.000	0.264	6.352	0.000	0.163	3.936	0.000	0.149	3.612	0.000
A/G FMR	-0.266	-7.683	0.000	-0.178	-4.694	0.000	-0.170	-4.471	0.000	-0.174	-4.626	0.000

Note: results expressed as standard *β* coefficients. A/G FMR: android/gynoid fat mass ratio; WBLM: whole-body lean mass; WBFM: whole-body fat mass; WB BMD: whole-body bone mineral density; LS BMD: lumbar spine bone mineral density; FN BMD: femoral neck bone mineral density; TH BMD: total hip bone mineral density.

## Data Availability

The data used to support the findings of this study are available from the corresponding author upon request.
